# Scrotal Pain after Varicocelectomy: A Narrative Review

**DOI:** 10.3390/biomedicines11041070

**Published:** 2023-04-01

**Authors:** Chien-Zhi Lai, Szu-Ju Chen, Chi-Ping Huang, Huey-Yi Chen, Ming-Yen Tsai, Po-Len Liu, Yung-Hsiang Chen, Wen-Chi Chen

**Affiliations:** 1 School of Medicine, College of Medicine, China Medical University, Taichung 404333, Taiwan; 2 Division of Urology, Department of Surgery, Taichung Veterans General Hospital, Taichung 407219, Taiwan; 3 Department of Urology, China Medical University Hospital, Taichung 404327, Taiwan; 4 Graduate Institute of Integrated Medicine, College of Chinese Medicine, China Medical University, Taichung 404333, Taiwan; 5 Department of Obstetrics and Gynecology, China Medical University Hospital, Taichung 404327, Taiwan; 6 Department of Chinese Medicine, Kaohsiung Chang Gung Memorial Hospital and Chang Gung University College of Medicine, Kaohsiung 833401, Taiwan; 7 Kaohsiung Municipal Feng Shan Hospital (Under the Management of Chang Gung Medical Foundation), Kaohsiung 830025, Taiwan; 8 Department of Respiratory Therapy, College of Medicine, Regenerative Medicine and Cell Therapy Research Center, Kaohsiung Medical University, Kaohsiung 807378, Taiwan; 9 Department of Psychology, College of Medical and Health Science, Asia University, Taichung 413305, Taiwan; 10 Department of Medical Research, China Medical University Hospital, Taichung 404327, Taiwan

**Keywords:** varicocelectomy, chronic scrotal pain, infertility, postoperative pain

## Abstract

Varicocele is a frequently encountered urological disorder, which has a prevalence rate of 8 to 15% among healthy men. However, the incidence is higher in male patients with primary or secondary infertility, with up to 35 to 80% of varicocele cases occurring in this population. The clinical manifestations of varicocele typically include the presence of an asymptomatic mass that feels like a “bag of worms”, chronic scrotal pain, and infertility. Most patients with varicocele only undergo varicocelectomy after conservative treatments have failed. Unfortunately, some patients may still experience persistent scrotal pain due to a recurrence of varicocele, the development of hydrocele, neuralgia, referred pain, ureteral lesions, or nutcracker syndrome. Therefore, clinicians should consider these conditions as potential causes of postoperative scrotal pain, and take measures to address them. Several factors can assist in predicting surgical outcomes for patients with varicocele. Clinicians should consider these factors when deciding whether to perform surgery and what type of surgical intervention to use. By doing so, they can increase the likelihood of a successful surgical outcome and minimize the risk of complications such as postoperative scrotal pain.

## 1. Introduction

Varicocele is a frequently diagnosed urological condition [1,2,3], affecting approximately 8 to 15% of healthy men [4,5]. The incidence of varicocele varies among different age groups. Varicocele was seldom observed in 2 to 10-year-old boys before puberty (1.75%) while the increasing frequency was noted in 11 to 14-year-old (7.8%) and 15 to 19-year-old (14.1%) boys [6]. It was reported by Levinger that the incidence of varicocele increases by about 10% with each decade of aging in men above 30 years, from 18% in the 30 to 40-year-old group to 75% in 80 to 90-year-old group [7]. In 465 patients over 40 years old, 48% of the incidence was described by Besiroglu in a cross-sectional study [8]. In addition, prevalence may have geographic differences in countries. Among 211,989 Chinese males of reproductive age and an average age of 31.20 years, a prevalence of 7.66% was observed [9]. There was an incidence of 15.7% in 7035 European young men with a median age of 19 years [10]. Carto et al. reported a 9.6% incidence rate in 101,309 male patients above 18 years with infertility collected from a US-based health research network [11]. Primary or secondary infertility accounts for the majority (35 to 80%) of varicocele cases in males [5,12]. In many cases, varicocele is asymptomatic, and only a proper clinical exam could assess the presence of varicocele. The clinical presentation of varicocele includes the presence of an asymptomatic mass that resembles a “bag of worms,” chronic scrotal discomfort, and infertility.

The venous drainage of the testes comprises a network of small spermatic veins that gradually coalesce to form a pampiniform plexus. This plexus runs alongside the testicular artery, and as it ascends through the inguinal canal, it unites with the testicular vein [13,14]. In addition to this main pathway, the pampiniform plexus also anastomoses with other collateral veins, such as the cremasteric veins, vasal veins, external pudendal veins, and gubernacular veins.

**Figure 1:**
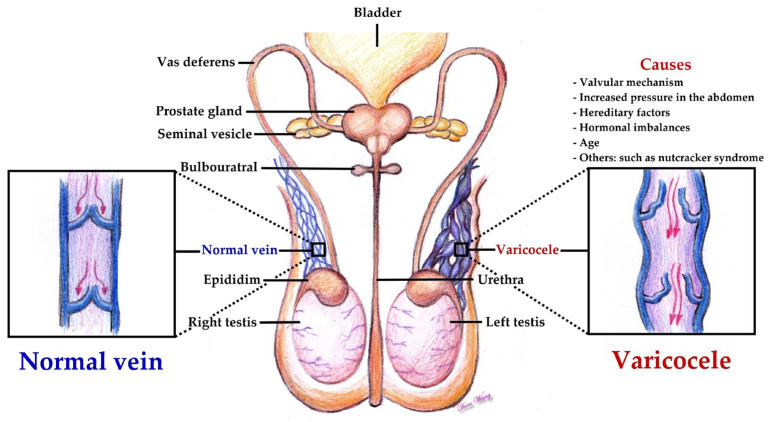
The scheme of pampiniform plexus; a loose network of small veins found within the male spermatic cord. The varicocele is a condition in which the veins in the scrotum, the loose pouch of skin that contains the testicles, become enlarged. These veins are responsible for carrying blood that has been depleted of oxygen away from the testicles. When blood begins to accumulate in the veins instead of flowing out of the scrotum, a varicocele develops.

Varicocele is a condition that arises when the pampiniform plexus becomes dilated and tortuous. This can result from various factors, such as increased venous pressure in the left renal or gonadal veins, reflux of collateral venous anastomoses, or the presence of incompetent or absent valves in the testicular veins (Figure 1). The incidence of varicocele in the general population is estimated to be around 15% [5], with most cases occurring on the left side, which was attributed to the anatomy of the perpendicular angle of the left gonadal vein and left renal vein and subsequent elevated venous pressure compared with the sharper angle of insertion between the right gonadal vein and inferior vena cava. If a varicocele presents only on the right side, other pathological causes should be considered, including renal or other retroperitoneal malignancy, which may cause venous obstruction and increase venous pressure on the right gonadal vein [15]. While some varicoceles may be asymptomatic, others can cause chronic scrotal pain, scrotal heaviness, and infertility. The incidence of pain among patients with varicocele is estimated to be between 2 and 10% [16]. Different types of pain have been reported, including dull, dragging, throbbing, and sharp pain. Varicocele may lead to a rise in intratesticular temperature, exposure to noxious insults, and varicocele-induced testicular defects, including decreased serum testosterone level. Therefore, varicoceles can further interfere with spermatogenesis and are recognized as the most common reversible cause of male infertility [17].

In this narrative review, we focused on the causes of pain following varicocelectomy. This surgical procedure is commonly performed to alleviate varicocele-related pain or infertility, and its success rate is generally high. However, some patients may experience pain or discomfort following the procedure, and identifying the underlying causes of this pain is essential for optimal management.

## 2. Diagnosis and Grading

### 2.1. Physical Examination

Physical examination is the cornerstone of diagnosing varicocele, and it involves a thorough evaluation of the scrotum and its content. To facilitate the examination, the patient is generally placed in a warm room to help relax the cremaster and dartos muscles, which can otherwise cause the scrotum to retract and make it difficult to visualize the veins. During the examination, the patient is usually evaluated in both standing and supine positions. The scrotum is exposed, and the examiner uses their fingers to palpate the spermatic cord, which should feel smooth and uniform in texture. In cases of varicocele, the veins in the pampiniform plexus will appear dilated and tortuous, giving the appearance of a “bag of worms”.

To enhance the visualization of the varicocele, the Valsalva maneuver is often employed. This involves the patient taking a deep breath and holding it while bearing down as if trying to have a bowel movement. This increases the intraabdominal and intrathoracic pressure, which reduces venous return to the heart and increases the peripheral venous volume, making the varicocele more apparent. In addition to the physical examination, imaging tests such as ultrasound or venography may also be used to confirm the diagnosis of varicocele, particularly in cases where the physical examination is equivocal or when the patient is obese. However, physical examination remains the most important method for diagnosing varicocele and guiding treatment decisions.

When diagnosing varicocele, it is essential to grade the severity of the condition to determine the most appropriate treatment plan. Since there are no universally accepted classification systems yet, several classification methods were proposed. In 1970, Dubin and Amelar described a grading scale as the following content with a patient in the upright position [18]. Grade 1 varicoceles are small (<1 cm) and palpable only during a Valsalva maneuver, while grade 2 varicoceles are moderate (1–2 cm) and easily palpable but not visible. Grade 3 varicoceles are large (>2 cm) and easily visible without the need for palpation. In 1994, Tauber proposed a classification based on Dubin and Amelar’s with Doppler examination and added Grade 0 varicoceles, which are subclinical, not palpable but can be detected under Doppler ultrasonography (Table 1) [19].

In addition to physical examination, imaging studies can also provide a more precise evaluation of varicocele. Ultrasonography is a noninvasive and widely used imaging technique that can accurately detect and grade varicocele. Sarteschi utilized ultrasonography to grade varicocele from grade 1 to 5 according to the severity of reflux, varicosities, and testicular atrophy [20]. Computed tomography (CT) and magnetic resonance imaging (MRI) are more advanced imaging techniques that can provide a clearer anatomical picture of the pelvic area, allowing for the detection of varicocele as well as other underlying conditions. Venography, which involves the injection of a contrast dye into the veins, is an invasive but highly accurate method for diagnosing varicocele and can be used to plan the optimal surgical approach.

Overall, the combination of physical examination and imaging studies can help diagnose and grade varicocele, allowing for appropriate treatment decisions to be made.

### 2.2. Treatments of Varicocele: Indications, Type of Surgery, and Its Results

#### 2.2.1. Conservative Treatments

Initially, for chronic pain, patients may be managed with conservative treatments, such as bed rest, scrotal elevation or support, perineal pelvic floor exercise [21], limitations on physical activities, and non-steroidal anti-inflammatory drugs (NSAIDs). Moreover, as per Kilic [22], it has been reported that phlebotrophic drugs such as the micronized purified flavonoid fraction (MPFF) can help to improve venous tone and elasticity and reduce vein distension. At the same time, it was not recommended to use MPFF as a conservative treatment, given that randomized placebo-controlled trials are not yet available.

In cases where conservative treatments are not effective, surgical intervention may be necessary. Surgical intervention is recommended mostly if there is a sperminogram impairment, and for patients experiencing persistent scrotal pain, testicular atrophy, and infertility despite receiving conservative treatment. This recommendation is supported by a retrospective review that showed that varicocele ligation was effective [16].

#### 2.2.2. Surgical treatments

Varicocelectomy

Retroperitoneal high ligation technique = Palomo approach

Varicocelectomy is a surgical procedure that involves ligating the dilated testicular veins to alleviate symptoms associated with varicocele (Table 2). The Palomo approach, first proposed by Palomo in 1949 [23], is a widely used technique for performing this procedure. It can be performed either openly, using a surgical microscope, or via laparoscopy. During the procedure, after accessing the retroperitoneal cavity, the testicular veins are ligated above the internal inguinal ring, with or without sparing the testicular artery. However, one limitation of the retroperitoneal approach is that it does not allow access to the collateral veins of the pampiniform plexus, which may lead to a higher recurrence rate [24,25]. Therefore, microsurgical subinguinal varicocelectomy, which allows for the identification and ligation of all dilated veins in the pampiniform plexus, has become a popular alternative. In addition to the risk of recurrence, the Palomo approach may also be associated with a lower pain remission rate compared to microsurgical subinguinal varicocelectomy [26]. Thus, it is essential to consider the patient’s specific circumstances and seek the opinion of an experienced surgeon when deciding on the most appropriate surgical approach.

Inguinal approach = Ivanissevich approach

Another approach for varicocelectomy is the inguinal approach, also known as the Ivanissevich approach. This technique involves making an incision in the external oblique aponeurosis to expose the inguinal canal, followed by dissection of the spermatic cord near the internal inguinal ring and ligation of the pampiniform plexus of veins. Despite its effectiveness, the inguinal approach carries the risk of damaging the ilioinguinal nerve and genital branch of the genitofemoral nerve, which can lead to chronic pain and numbness in the groin area [26]. Moreover, this approach may increase the risk of herniation due to the weakening of the abdominal wall. Additionally, opening the external oblique aponeurosis may increase the incidence of postoperative pain and prolong recovery time. As a result, while the inguinal approach may be effective in certain cases, it is generally considered less favorable than microsurgical subinguinal varicocelectomy, which has a lower risk of complications and offers comparable success rates. Ultimately, the choice of surgical approach will depend on the individual patient’s medical history, physical examination findings, and surgeon preference.

Subinguinal approach = Goldstein approach

One of the most widely used techniques for varicocelectomy is the subinguinal approach. This procedure involves the dissection of the spermatic cord inferior to the external inguinal ring while maintaining an intact external oblique aponeurosis. The microanatomy of this approach involves the identification and ligation of more small internal spermatic veins and fewer large internal spermatic veins, with a greater number of external spermatic veins requiring ligation compared to the inguinal approach. Furthermore, the presence of more veins surrounding the spermatic arteries makes the subinguinal approach more challenging, requiring longer surgical time and a higher level of technical skill [27,28]. Despite the technical challenges, the subinguinal approach has become the preferred method for varicocelectomy due to its many advantages. These include less postoperative pain, shorter recovery time, and a lower rate of recurrence compared to other approaches [29]. Furthermore, the preservation of the external oblique aponeurosis reduces the risk of postoperative hernia and abdominal wall weakness, making this approach ideal for patients who are at higher risk for these complications. Therefore, the subinguinal approach has emerged as the mainstay of varicocelectomy due to its many advantages, including a lower rate of recurrence, shorter recovery time, and less postoperative pain. However, the choice of surgical approach should always be tailored to the individual patient, taking into account the surgeon’s experience and the patient’s unique anatomy and medical history.

Laparoscopic transperitoneal intra-abdominal approach

Laparoscopic varicocelectomy aims to ligate the gonadal veins above the internal inguinal ring with intact gonadal arteries. Thus, surgeons can perform bilateral varicocelectomy sequentially to magnify the testicular vessels, which improves effectiveness [26].

Microscopic varicocelectomy was found to be superior to the laparoscopic approach in terms of surgical outcomes (*p* < 0.0001) [30]. Furthermore, due to its inability to reach the external gonadal vessels or external spermatic veins [31], laparoscopic varicocelectomy was reported to have a higher recurrence rate (17.2%, 34 of 198 patients) compared with microsurgical and open nonmicrosurgical methods [32].

Percutaneous embolization = endovascular approach

Percutaneous embolization can be performed through the puncture of the internal jugular vein (antegrade) or common femoral vein (retrograde) to access the renal and internal spermatic veins with an appropriate catheter. The sources of occlusion may be sclerosants, solid embolic devices [33], or balloon embolotherapy [34]. As a minimally invasive procedure, in addition to the high success rate (95.7%, 68 of 71 patients) and low recurrence rate (1.96%) as reported by Nabi in 2004 [35], percutaneous embolization plays an important role in the treatment of varicoceles.

**Table 2:** Surgical treatments for varicocele.

Varicocelectomy	Patients (n)	Rate of Pain Relief		Recurrence or Failure	References
		Partial	Complete		
Retroperitoneal	37	32/37 (86.5%)		5/37 (13.5%)	[36]
	87	8/87 (9.2%)	72/87 (82.8%)	7/87 (8%)	[37]
Inguinal	114	104/114 (91.2%)		10/114 (8.8%)	[38]
	44	18/44 (40.9%)	24/44 (54.5%)	2/44 (4.6%)	[39]
Sub-inguinal	9	4/9 (44.4%)	4/9 (44.4%)	1/9 (11.1%)	[39]
	34	30/34 (89.5%)		4/34 (10.5%)	[40]
	81	16/81 (19.8%)	58/81 (71.6%)	7/81 (8.6%)	[41]
	132	110/132 (83.3%)		22/132 (16.7%)	[42]
	237	15/237 (6.3%)	203/237 (85.6%)	19/237 (8.1%)	[43]
Laparoscopic	60	16/60 (26.7%)	27/60 (45%)	17/60 (28.3%)	[44]
	48	5/48 (10.4%)	42/48 (87.5%)	1/48 (2%)	[45]
Percutaneous embolization	71	68/71 (95.8%)		3/71 (4.2%)	[35]

Causes of persistent scrotal pain after treatment: recurrent, neuralgia, refer pain, hydrocele, ureteral lesion, nutcracker syndrome, and unknown.

## 3. Recurrence

Despite advances in surgical techniques, recurrent varicoceles remain a common cause of persistent pain after treatment, and the recurrence rate can vary significantly among different surgical approaches. Several factors have been suggested to be associated with recurrence, including the patient’s internal spermatic veins and collateral veins [46]. To accurately evaluate varicocele recurrence and determine the appropriate course of treatment, ultrasonography is recommended as the primary diagnostic tool. In cases of recurrent varicoceles, treatment options may include angiographic embolization or repeat surgery. Reports indicate that microscope-assisted a subinguinal varicocelectomy with delivery to the testis is the optimal treatment for recurrent varicocele, with a significant decrease in the recurrence rate [46]. Microscopic subinguinal varicocelectomy is reported to have a lower recurrence rate than other approaches due to the ability to identify and ligate more dilated veins under a larger magnification of the surgical field [32,40]. Additionally, the subinguinal approach has the advantage of preserving the external oblique aponeurosis, reducing the risk of postoperative hernia and abdominal wall weakness. However, the choice of surgical approach should always be individualized to the patient [47], considering the surgeon’s experience, the patient’s unique anatomy, and medical history.

Recurrent varicoceles can be challenging to manage and require careful evaluation and individualized treatment planning. Ultrasonography is recommended for the evaluation of recurrence, and microscopic subinguinal varicocelectomy with delivery to the testis is an optimal treatment option with a low recurrence rate. However, the choice of surgical approach should always be tailored to the individual patient, and additional research is needed to evaluate further the long-term outcomes of different surgical techniques for recurrent varicoceles.

## 4. Neuralgia

Patients who complained of scrotal pain for >3 months with both a sonogram and an upper urinary tract survey producing negative results were defined as having chronic scrotal content pain [48]. Chronic scrotal content pain was speculated to result from Wallerian degeneration of nerves innervating the cremaster muscle, vasal sheath, perivasal tissue, and posterior cord lipomatous tissue under the study of spermatic cord biopsy [49,50]. Potential conservative treatments include pelvic floor therapy, tricyclic antidepressants (TCAs), gabapentin, NSAIDs, and muscle relaxants [21,51].

Spermatic cord block with an injection of a local anesthetic can temporarily alleviate scrotal pain, while microsurgical denervation of the spermatic cord (MDSC) and targeted denervation of the spermatic cord (TMDSC) using a subinguinal approach are both options for surgical management. Before performing MDSC or TMDSC, it is necessary to perform a spermatic cord block to evaluate pain relief and predict the success of these procedures [21,52]. MDSC requires the ligation of all spermatic cord structures with the exception of the arteries, lymphatics, and vas through the subinguinal approach. In contrast, TMDSC, a simplification of MDSC, involves the ligation of the aforementioned Wallerian degeneration areas: the cremaster muscle, vasal sheath, peri-vasal tissue, and posterior cord lipomatous tissue, which maintains the internal spermatic cord intact and requires shorter surgical time. A comparison of these two denervation procedures revealed that MDSC required a longer operative time than TMDSC (53 min in MDSC versus 21 min in TMDSC) [53] with similar successful pain-relieving rates (82.1–92.1% in MDSC versus 83–93% in TMDSC, Table 3).

Stimulation of the peripheral nerves or the spinal cord [54,55,56] and cryoablation of tissue innervation around the spermatic cord have been reported to relieve chronic scrotal pain [57].

**Table 3:** Successful pain relieving rates of MDSC and TMDSC.

References	Type of Denervation	Number of Units and Patients	Rate of Pain Relieving	Follow-up Period
Long, et al. [58]	MDSC	28 units in 28 patients	82.1%	1 year
Levine, et al. [59]	MDSC	33 units in 27 patients	84.8%	24 months
Oomen, et al. [60]	MDSC	58 units in 51 patients	86.2%	42.8 months
Strom, et al. [61]	MDSC	95 units in 79 patients	88.4%	20.3 months
Chaudhari, et al. [62]	MDSC	38 units in 31 patients	92.1%	2 years
Kavoussi [53]	MDSC	39 units in 39 patients	84.6%	1 year
	TMDSC	43 units in 43 patients	93.0%	1 year
Kavoussi [63]	TMDSC	25 units in 19 patients	92.0%	6 months
Calixte, et al. [64]	TMDSC	860 units in 772 patients	83.0%	4 years

MDSC: Microsurgical denervation of the spermatic cord, TMDSC: targeted denervation of the spermatic cord.

## 5. Referred Pain

Several urological conditions could result in referred pain in the scrotal area, such as chronic prostatitis and orchitis [65,66]. The scrotum is innervated anteriorly by the anterior scrotal nerve branched from the ilioinguinal nerve and the genital branch of the genitofemoral nerve, posteriorly by the posterior scrotal nerve, and inferiorly by the perineal branch of the posterior cutaneous nerve of the thigh [67]. Thus, the origins of pain, such as abdominal aortic aneurysm, urolithiasis, prolapse of the intervertebral disc, retrocecal appendicitis, impingement of the nerve root, pudendal neuropathy, and retroperitoneal neoplasm [68], which share the same pathway as the aforementioned nerves, may result in scrotal referred pain.

Additionally, because the testis, prostate, and kidney share overlapping autonomic innervation, chronic prostatitis, orchitis, and urologic lesions may present radiation pain at the groin and scrotum. Innervation of the ureter branch from the autonomic plexuses, which are in close proximity (renal, aortic, superior hypogastric, and inferior hypogastric plexuses), which contain mixed sympathetic and parasympathetic nerves. Ureter pain is transmitted along the sympathetic nerve fibers to the T10–L2 spinal cord in a retrograde manner. Lesions in the ureter, such as ureteric calculi and neoplasms, can cause colicky pain in the lower abdomen when a part of the ureter is superior to the site obstructed by the stone. Additionally, ureteric pain may generate referred pain at the ipsilateral flank and groin region, which is correlated with the dermatome of T10–L2. Consequently, ureteral lesions may be a cause of scrotal pain.

Chronic inflammatory epididymitis is a potential cause of postoperative scrotal pain resulting from infection after surgery or previous viral or bacterial infections. Interstitial cystitis, also known as bladder pain syndrome of unknown etiology, is a possible cause of scrotal pain accompanied by pelvic pain and lower urinary tract symptoms [69].

## 6. Hydrocele

Interference with testicular lymphatic drainage during varicocelectomy has been reported to cause postoperative hydroceles. According to a systemic review in 2018, the average percentage of postoperative hydrocele was 0.6% in the microsurgical subinguinal approach, followed by 5.3% in the open inguinal, 6.7% in the laparoscopic, and 7.5% in the retroperitoneal approach [26]. Hydroceles present as an accumulation of transilluminated fluid between the visceral and parietal layers of the tunica vaginalis. Hydroceles may result from an imbalance of fluid production and absorption. Lymphatic stomata on the parietal layer of the tunica vaginalis have been reported by Wang et al. [70]. Accordingly, the cavity of the tunica vaginalis is connected to the testicular lymphatic system through lymphatic stomata and testicular lymphatic drainage up to the lumbar and para-aortic nodes, which may explain the postoperative hydrocele observed following varicocelectomy. With the assistance of microsurgery or lymphatic staining, the lymphatic channels can be magnified and visualized, reducing the risk of hydrocele formation [24,27,71].

## 7. Nutcracker Syndrome

Nutcracker syndrome, also called anterior nutcracker syndrome, generally presents as left renal vein entrapment syndrome, which refers to the entrapment of the left renal vein between the superior mesenteric artery (SMA) and abdominal aorta. The SMA arises from the abdominal aorta at the T1 level, and the angle between the SMA and the abdominal aorta is frequently <35° in patients with nutcracker syndrome. This narrow angle leads to higher pressure in the left renal vein causing dilatation of the left gonadal vein and communicating lumbar vein. In addition to anterior nutcracker syndrome, there is a 0.1–3.2% [72] incidence of retroaortic or circumaortic left renal vein due to venous anomalies, which may lead to compression of retroaortic or circumaortic left renal vein between the abdominal aorta and vertebral column, known as posterior nutcracker syndrome. Venous hypertension may also cause rupture of the small veins in the renal fornix. Hematuria, left flank pain, and varicocele are the three typical clinical features of nutcracker syndrome [73]. Other manifestations include orthostatic proteinuria, abdominal pain, left loin pain, scrotal discomfort, dysuria, and tachycardia [74]. Owing to these nonspecific symptoms, nutcracker syndrome is underdiagnosed. Tall and thin patients are more vulnerable to nutcracker syndrome because the angle between the SMA and abdominal aorta is more acute [75].

Nutcracker syndrome can be diagnosed as a pressure gradient of >2 mmHg [76] between the renal vein and the inferior vena cava via retrograde venography. Because of its noninvasiveness and better accessibility, computed tomography angiography (CTA) allows for more detailed anatomy and evaluation of the venous system, with “the beak sign” at the narrowing portion of the left renal vein observed on axial CT [77].

Treatments for nutcracker syndrome are controversial. Conservative management includes weight gain and medical therapy such as angiotensin converting enzyme inhibitors and aspirin for orthostatic proteinuria and improving renal perfusion, respectively [78]. If patients present with gross hematuria and severe symptoms with conservative vein treatment, surgical intervention is indicated. Open approaches include renal autotransplantation, left renal vein transposition, and mesoaortic transposition [77]. Laparoscopic left renal vein transposition, endovascular stenting of the left renal vein, gonadocava bypass, and transluminal balloon angioplasty are all less invasive surgical treatments. As nutcracker syndrome is often misdiagnosed, this disease should be considered when encountering hematuria of unknown origin.

## 8. Predictors of Treatment Outcome

Indicators, including a higher preoperative grade of varicocele [41], pain characteristics, duration of pain before surgery, surgical approach, a greater number of ligated veins [79], diameter of the dilated veins, younger age, and body mass index (BMI), have been proposed to be associated with better surgical outcomes for painful varicocele.

A retrospective study in 2020 investigated the factors contributing to the recurrence of varicocele in 34 patients after microscopic subinguinal varicocelectomy, revealing that a higher grade of varicocele on the left side (*p* = 0.024) and larger diameter of dilated veins preoperatively (*p* = 0.002) were significantly associated with recurrence [40]. The presentation of dull pain has been reported to be a predictor of better outcomes, with a 100% success rate in the dull pain group [24,38]. Park et al. reported that a shorter time span from varicocele pain onset to surgery (<6 months) resulted in greater improvement in pain resolution (*p* = 0.004) [39]. However, some studies revealed no significant differences between the duration of preoperative pain and pain relief [24,42].

Park and Gorur also reported that a higher BMI was significantly associated with pain improvement [80]. However, differences in surgical approaches may also affect the outcomes. A systematic review of 36 studies [31] and a retrospective cohort study [40] concluded that open microsurgical subinguinal or inguinal varicocelectomy had the lowest recurrence rate of 1.05% (0.00–3.57%). Regarding these indicators, urologists should be aware of them and carefully evaluate patients before varicocelectomy and during postoperative follow-up.

## 9. Conclusions

This review discusses the pain relieving rates of several surgical approaches, predictors of surgical outcomes, and some conditions that may lead to persistent scrotal pain after varicocelectomy. However, this article is subject to some potential limitations; there are no standard databases and organized methods to analyze data and collect studies related to varicocele for this review. The extent of reviewed articles was also limited and may be unable to illustrate all possible causes of postoperative scrotal pain. Pathophysiology of varicocele was not discussed thoroughly in this article as well. In summary, surgical treatment for varicocele can be effective in relieving pain and improving fertility, and the choice of surgical approach should be based on individual patient factors. Predictors of surgical outcomes should be taken into consideration, and careful evaluation is necessary in cases of persistent pain after surgery to identify the underlying cause and provide appropriate management.

## Figures and Tables

**Table 1:** Classification of varicocele.

Dubin and Amelar; Tauber	**Grade**	**Clinical**	**Vein Convolution**	**Nomenclature**
	0	Not palpable but can be detected under Doppler ultrasonography	0	Subclinical
	1	Palpable only during a Valsalva maneuver	0–1 cm	Small
	2	Easily palpable but not visible	1–2 cm	Moderate
	3	Easily visible without the need for palpation	>2 cm	Large
Sarteschi	**Grade**	**Reflux**	**Varicosities**	**Testicular Hypotrophy**
	1	During a Valsalva maneuver	None	No
	2	During a Valsalva maneuver	Small	No
	3	Obvious during a Valsalva maneuver	Apparent	No
	4	Spontaneous reflux and increase during a Valsalva maneuver or standing	Present in all positions	Common
	5	Spontaneous reflux at rest without increases during a Valsalva maneuver	Venous dilatation in all positions	Yes

## Data Availability

All data are available upon request to the corresponding author.

## References

[1] Bebi C., Bilato M., Minoli D.G., De Marco E.A., Gnech M., Paraboschi I., Boeri L., Fulgheri I., Brambilla R., Campoleoni M. (2023). Radiation Exposure and Surgical Outcomes after Antegrade Sclerotherapy for the Treatment of Varicocele in the Paediatric Population: A Single Centre Experience. J. Clin. Med..

[2] Chevallier O., Fauque P., Poncelet C., Guillen K., Comby P.O., Astruc K., Barberet J., Falvo N., Simon E., Loffroy R. (2021). Relevant Biological Effects of Varicocele Embolization with N-Butyl Cyanoacrylate Glue on Semen Parameters in Infertile Men. Biomedicines.

[3] Wang X., Pan C., Li J., Zhan Y., Liu G., Bai S., Chai J., Shan L. (2022). Prospective Comparison of Local Anesthesia with General or Spinal Anesthesia in Patients Treated with Microscopic Varicocelectomy. J. Clin. Med..

[4] Clarke B.G. (1966). Incidence of Varicocele in Normal Men and Among Men of Different Ages. JAMA.

[5] Alsaikhan B., Alrabeeah K., Delouya G., Zini A. (2016). Epidemiology of varicocele. Asian J. Androl..

[6] Akbay E., Cayan S., Doruk E., Duce M.N., Bozlu M. (2000). The prevalence of varicocele and varicocele-related testicular atrophy in Turkish children and adolescents. BJU Int..

[7] Levinger U., Gornish M., Gat Y., Bachar G.N. (2007). Is varicocele prevalence increasing with age?. Andrologia.

[8] Besiroglu H., Otunctemur A., Dursun M., Ozbek E. (2019). The prevalence and severity of varicocele in adult population over the age of forty years old: A cross-sectional study. Aging Male.

[9] Hu X., Yang X., Zhao J., Guan T., Dai Q., Yang J., Zhang H., Zhang D., Zhang Y., Shang L. (2022). Association between body mass index and varicocele among 211 989 Chinese reproductive-age males. Int. J. Urol..

[10] Damsgaard J., Joensen U.N., Carlsen E., Erenpreiss J., Blomberg Jensen M., Matulevicius V., Zilaitiene B., Olesen I.A., Perheentupa A., Punab M. (2016). Varicocele Is Associated with Impaired Semen Quality and Reproductive Hormone Levels: A Study of 7035 Healthy Young Men from Six European Countries. Eur. Urol..

[11] Carto C., Gandhi D.A., Nackeeran S., Madhusoodanan V., Ramasamy R. (2022). Varicocele is underdiagnosed in men evaluated for infertility: Examination of multi-center large-scale electronic health record data. Andrologia.

[12] Clavijo R.I., Carrasquillo R., Ramasamy R. (2017). Varicoceles: Prevalence and pathogenesis in adult men. Fertil. Steril..

[13] Naughton C.K., Nangia A.K., Agarwal A. (2001). Pathophysiology of varicoceles in male infertility. Hum. Reprod. Update.

[14] Hutson J.M., Coran A.G. (2012). Chapter 77—Undescended Testis, Torsion, and Varicocele. Pediatric Surgery.

[15] Gleason A., Bishop K., Xi Y., Fetzer D.T. (2019). Isolated Right-Sided Varicocele: Is Further Workup Necessary?. Am. J. Roentgenol..

[16] Peterson A.C., Lance R.S., Ruiz H.E. (1998). Outcomes of varicocele ligation done for pain. J. Urol..

[17] Cozzolino D.J., Lipshultz L.I. (2001). Varicocele as a progressive lesion: Positive effect of varicocele repair. Hum. Reprod. Update.

[18] Dubin L., Amelar R.D. (1970). Varicocele Size And Results of Varicocelectomy in Selected Subfertile Men with Varicocele*. Fertil. Steril..

[19] Tauber R., Johnsen N. (1994). Antegrade scrotal sclerotherapy for the treatment of varicocele: Technique and late results. J. Urol..

[20] Sarteschi M. (1993). Lo studio del varicocele con eco-color-Doppler. G It Ultrason.

[21] Malaguti S.A., Lund L. (2021). Gold Standard Care of Chronic Scrotal Pain. Res. Rep. Urol..

[22] Kiliç S., Güneş A., Ipek D., Dusak A., Güneş G., Balbay M.D., Baydinç Y.C. (2005). Effects of micronised purified flavonoid fraction on pain, spermiogram and scrotal color Doppler parameters in patients with painful varicocele. Urol. Int..

[23] Palomo A. (1949). Radical cure of varicocele by a new technique; preliminary report. J. Urol..

[24] Paick S., Choi W.S. (2019). Varicocele and Testicular Pain: A Review. World J. Mens. Health.

[25] Tsai C.S., Lin F.Y., Chen Y.H., Yang T.L., Wang H.J., Huang G.S., Lin C.Y., Tsai Y.T., Lin S.J., Li C.Y. (2008). Cilostazol attenuates MCP-1 and MMP-9 expression in vivo in LPS-administrated balloon-injured rabbit aorta and in vitro in LPS-treated monocytic THP-1 cells. J. Cell. Biochem..

[26] Lundy S.D., Sabanegh E.S. (2018). Varicocele management for infertility and pain: A systematic review. Arab. J. Urol..

[27] Hopps C.V., Lemer M.L., Schlegel P.N., Goldstein M. (2003). Intraoperative varicocele anatomy: A microscopic study of the inguinal versus subinguinal approach. J. Urol..

[28] Liu W.L., Chang J.M., Chong I.W., Hung Y.L., Chen Y.H., Huang W.T., Kuo H.F., Hsieh C.C., Liu P.L. (2017). Curcumin Inhibits LIN-28A through the Activation of miRNA-98 in the Lung Cancer Cell Line A549. Molecules.

[29] Owen R.C., McCormick B.J., Figler B.D., Coward R.M. (2017). A review of varicocele repair for pain. Transl. Androl. Urol..

[30] Park J.H., Pak K., Park N.C., Park H.J. (2021). How Can We Predict a Successful Outcome after Varicocelectomy in Painful Varicocele Patients? An Updated Meta-Analysis. World J. Mens. Health.

[31] Rotker K., Sigman M. (2015). Recurrent varicocele. Asian J. Androl..

[32] Ding H., Tian J., Du W., Zhang L., Wang H., Wang Z. (2012). Open non-microsurgical, laparoscopic or open microsurgical varicocelectomy for male infertility: A meta-analysis of randomized controlled trials. BJU Int..

[33] JR D.B. (2007). Percutaneous varicocele embolization. Can. Urol. Assoc. J..

[34] Shuman L., White R.I., Mitchell S.E., Kadir S., Kaufman S.L., Chang R. (1986). Right-sided varicocele: Technique and clinical results of balloon embolotherapy from the femoral approach. Radiology.

[35] Nabi G., Asterlings S., Greene D.R., Marsh R.L. (2004). Percutaneous embolization of varicoceles: Outcomes and correlation of semen improvement with pregnancy. Urology.

[36] Abd Ellatif M.E., Asker W., Abbas A., Negm A., Al-Katary M., El-Kaffas H., Moatamed A. (2012). Varicocelectomy to treat pain, and predictors of success: A prospective study. Curr. Urol..

[37] Yeniyol C.O., Tuna A., Yener H., Zeyrek N., Tilki A. (2003). High ligation to treat pain in varicocele. Int. Urol. Nephrol..

[38] Kim H.T., Song P.H., Moon K.H. (2012). Microsurgical ligation for painful varicocele: Effectiveness and predictors of pain resolution. Yonsei Med. J..

[39] Park H.J., Lee S.S., Park N.C. (2011). Predictors of pain resolution after varicocelectomy for painful varicocele. Asian J. Androl..

[40] Alkhamees M., Bin Hamri S., Alhumaid T., Alissa L., Al-Lishlish H., Abudalo R., Iqbal Z., Albajhan G., Alasker A. (2020). Factors Associated with Varicocele Recurrence After Microscopic Sub-Inguinal Varicocelectomy. Res. Rep. Urol..

[41] Kim S.-O., Jung H., Park K. (2012). Outcomes of Microsurgical Subinguinal Varicocelectomy for Painful Varicoceles. J. Androl..

[42] Al-Gadheeb A., El-Tholoth H.S., Albalawi A., Althobity A., AlNumi M., Alafraa T., Jad A. (2021). Microscopic subinguinal varicocelectomy for testicular pain: A retrospective study on outcomes and predictors of pain relief. Basic Clin. Androl..

[43] Altunoluk B., Soylemez H., Efe E., Malkoc O. (2010). Duration of preoperative scrotal pain may predict the success of microsurgical varicocelectomy. Int. Braz. J. Urol..

[44] Park Y.W., Lee J.H. (2013). Preoperative Predictors of Varicocelectomy Success in the Treatment of Testicular Pain. World J. Mens. Health.

[45] Kachrilas S., Popov E., Bourdoumis A., Akhter W., El Howairis M., Aghaways I., Masood J., Buchholz N. (2014). Laparoscopic varicocelectomy in the management of chronic scrotal pain. JSLS J. Soc. Laparoendosc. Surg..

[46] Moon K.H., Cho S.J., Kim K.S., Park S., Park S. (2012). Recurrent varicoceles: Causes and treatment using angiography and magnification assisted subinguinal varicocelectomy. Yonsei Med. J..

[47] Chen W.C., Wu S.Y., Liu H.P., Chang C.H., Chen H.Y., Chen H.Y., Tsai C.H., Chang Y.C., Tsai F.J., Man K.M. (2010). Identification of melamine/cyanuric acid-containing nephrolithiasis by infrared spectroscopy. J. Clin. Lab. Anal..

[48] Ziegelmann M.J., Farrell M.R., Levine L.A. (2019). Evaluation and Management of Chronic Scrotal Content Pain-A Common Yet Poorly Understood Condition. Rev. Urol..

[49] Huang W.T., Chong I.W., Chen H.L., Li C.Y., Hsieh C.C., Kuo H.F., Chang C.Y., Chen Y.H., Liu Y.P., Lu C.Y. (2019). Pigment epithelium-derived factor inhibits lung cancer migration and invasion by upregulating exosomal thrombospondin 1. Cancer Lett..

[50] Parekattil S.J., Gudeloglu A., Brahmbhatt J.V., Priola K.B., Vieweg J., Allan R.W. (2013). Trifecta nerve complex: Potential anatomical basis for microsurgical denervation of the spermatic cord for chronic orchialgia. J. Urol..

[51] Chen J.W., Lin F.Y., Chen Y.H., Wu T.C., Chen Y.L., Lin S.J. (2004). Carvedilol inhibits tumor necrosis factor-alpha-induced endothelial transcription factor activation, adhesion molecule expression, and adhesiveness to human mononuclear cells. Arterioscler. Thromb. Vasc. Biol..

[52] Parekattil S.J. (2020). Targeted Microsurgical Denervation of the Spermatic Cord for Chronic Orchialgia: The Current Standard of Care. Curr. Urol. Rep..

[53] Kavoussi P.K. (2019). Validation of targeted microsurgical spermatic cord denervation: Comparison of outcomes to traditional complete microsurgical spermatic cord denervation. Asian J. Androl..

[54] Rosendal F., Moir L., de Pennington N., Green A.L., Aziz T.Z. (2013). Successful treatment of testicular pain with peripheral nerve stimulation of the cutaneous branch of the ilioinguinal and genital branch of the genitofemoral nerves. Neuromodulation.

[55] Paolini F., Ferini G., Bonosi L., Costanzo R., Brunasso L., Benigno U.E., Porzio M., Gerardi R.M., Giammalva G.R., Umana G.E. (2022). Spinal Cord Stimulation to Treat Unresponsive Cancer Pain: A Possible Solution in Palliative Oncological Therapy. Life.

[56] Parekattil S.J., Ergun O., Gudeloglu A. (2020). Management of Chronic Orchialgia: Challenges and Solutions—The Current Standard of Care. Res. Rep. Urol..

[57] Calixte N., Kartal I.G., Tojuola B., Gudeloglu A., Etafy M., Brahmbhatt J.V., Mendelson R.A., Chetta M., Parekattil S.J. (2019). Salvage Ultrasound-guided Targeted Cryoablation of The Perispermatic Cord For Persistent Chronic Scrotal Content Pain After Microsurgical Denervation Of The Spermatic Cord. Urology.

[58] Long H., Bai W., Zhang X., Xu T. (2019). A Clinical Study on Microsurgical Denervation of Spermatic Cord for Refractory Chronic Orchialgia. Urol. Int..

[59] Levine L.A., Matkov T.G. (2001). Microsurgical denervation of the spermatic cord as primary surgical treatment of chronic orchialgia. J. Urol..

[60] Oomen R.J.A., Witjens A.C., van Wijck A.J.M., Grobbee D.E., Lock T. (2014). Prospective double-blind preoperative pain clinic screening before microsurgical denervation of the spermatic cord in patients with testicular pain syndrome. Pain.

[61] Strom K.H., Levine L.A. (2008). Microsurgical denervation of the spermatic cord for chronic orchialgia: Long-term results from a single center. J. Urol..

[62] Chaudhari R., Sharma S., Khant S., Raval K. (2019). Microsurgical Denervation of Spermatic Cord for Chronic Idiopathic Orchialgia: Long-Term Results from an Institutional Experience. World J. Mens. Health.

[63] Kavoussi P.K. (2020). Microsurgical subinguinal cremaster muscle release for chronic orchialgia secondary to hyperactive cremaster muscle reflex in adults. Andrologia.

[64] Calixte N., Tojuola B., Kartal I., Gudeloglu A., Hirsch M., Etafy M., Mendelson R., Djokic B., Sherba S., Shah K. (2018). Targeted Robotic Assisted Microsurgical Denervation of the Spermatic Cord for the Treatment of Chronic Orchialgia or Groin Pain: A Single Center, Large Series Review. J. Urol..

[65] Crocetto F., Barone B., De Luca L., Creta M. (2020). Granulomatous prostatitis: A challenging differential diagnosis to take into consideration. Future Oncol..

[66] Crocetto F., Arcaniolo D., Napolitano L., Barone B., La Rocca R., Capece M., Caputo V.F., Imbimbo C., De Sio M., Calace F.P. (2021). Impact of Sexual Activity on the Risk of Male Genital Tumors: A Systematic Review of the Literature. Int. J. Environ. Res. Public Health.

[67] Patel A.P. (2017). Anatomy and physiology of chronic scrotal pain. Transl. Androl. Urol..

[68] McGee S.R. (1993). Referred scrotal pain: Case reports and review. J. Gen. Intern. Med..

[69] Akiyama Y., Luo Y., Hanno P.M., Maeda D., Homma Y. (2020). Interstitial cystitis/bladder pain syndrome: The evolving landscape, animal models and future perspectives. Int. J. Urol..

[70] Wang J., Ping Z., Jiang T., Yu H., Wang C., Chen Z., Zhang X., Xu D., Wang L., Li Z. (2013). Ultrastructure of lymphatic stomata in the tunica vaginalis of humans. Microsc. Microanal..

[71] (2007). Lymphatic staining to prevent hydrocele after laparoscopic varicocele ligation. Nat. Clin. Pract. Urol..

[72] Knipp B., Knechtges P., Gest T., Wakefield T. (2009). Inferior Vena Cava: Embryology and Anomalies. Aortic Aneurysms.

[73] Orczyk K., Wysiadecki G., Majos A., Stefańczyk L., Topol M., Polguj M. (2017). What Each Clinical Anatomist Has to Know about Left Renal Vein Entrapment Syndrome (Nutcracker Syndrome): A Review of the Most Important Findings. Biomed. Res. Int..

[74] Grimm L.J., Engstrom B.I., Nelson R.C., Kim C.Y. (2013). Incidental detection of nutcracker phenomenon on multidetector CT in an asymptomatic population: Prevalence and associated findings. J. Comput. Assist. Tomogr..

[75] Shaper K.R., Jackson J.E., Williams G. (1994). The nutcracker syndrome: An uncommon cause of haematuria. Br. J. Urol..

[76] Gulleroglu K., Gulleroglu B., Baskin E. (2014). Nutcracker syndrome. World J. Nephrol..

[77] Kim K.W., Cho J.Y., Kim S.H., Yoon J.H., Kim D.S., Chung J.W., Park J.H. (2011). Diagnostic value of computed tomographic findings of nutcracker syndrome: Correlation with renal venography and renocaval pressure gradients. Eur. J. Radiol..

[78] Ananthan K., Onida S., Davies A.H. (2017). Nutcracker Syndrome: An Update on Current Diagnostic Criteria and Management Guidelines. Eur. J. Vasc. Endovasc. Surg..

[79] Chen S.S. (2012). Factors predicting symptomatic relief by varicocelectomy in patients with normospermia and painful varicocele nonresponsive to conservative treatment. Urology.

[80] Gorur S., Candan Y., Helli A., Akcin S., Cekirge S.D., Kaya Y.S., Cekic C., Kiper A.N. (2015). Low body mass index might be a predisposing factor for varicocele recurrence: A prospective study. Andrologia.

